# In Absentia: An Exploratory Study of How Patients Are Considered in Multidisciplinary Cancer Team Meetings

**DOI:** 10.1371/journal.pone.0139921

**Published:** 2015-10-06

**Authors:** Pola Hahlweg, Jana Hoffmann, Martin Härter, Dominick L Frosch, Glyn Elwyn, Isabelle Scholl

**Affiliations:** 1 Department of Medical Psychology, University Medical Center Hamburg-Eppendorf, Hamburg, Germany; 2 Gordon and Betty Moore Foundation, Palo Alto, California, United States of America; 3 Department of Medicine, University of California Los Angeles, Los Angeles, California, United States of America; 4 The Dartmouth Institute for Health Policy and Clinical Practice, Dartmouth College, Hanover, New Hampshire, United States of America; University of Stirling, UNITED KINGDOM

## Abstract

**Background:**

Multidisciplinary team meetings and shared decision-making are potential means of delivering patient-centred care. Not much is known about how those two paradigms fit together in cancer care. This study aimed to investigate how decisions are made in multidisciplinary team meetings and whether patient perspectives are incorporated in these decisions.

**Materials and Methods:**

A qualitative study was conducted using non-participant observation at multidisciplinary team meetings (also called tumor boards) at the University Cancer Center Hamburg-Eppendorf, Germany. Two researchers recorded structured field notes from a total of N = 15 multidisciplinary team meetings. Data were analyzed using content analysis and descriptive statistics.

**Results:**

Physicians mainly exchanged medical information and based their decision-making on this information. Individual patient characteristics or their treatment preferences were rarely considered or discussed. In the few cases where patient preferences were raised as a topic, this information did not seem to be taken into account in decision-making processes about treatment recommendations.

**Conclusion:**

The processes in multidisciplinary team meetings we observed did not exhibit shared decision-making. Patient perspectives were absent. If multidisciplinary team meetings wish to become more patient-centred they will have to modify their processes and find a way to include patient preferences into the decision-making process.

## Introduction

Multidisciplinary care has become central to high quality cancer care, supported by many oncological associations and national cancer control plans [1, 2]. For many tumours, a growing number of potentially viable treatment options exist, offered by different health-care disciplines (e.g., medical versus radiation oncology versus surgical interventions). Thus, in order to be able to offer high quality clinical care, it is necessary to bring together multiple professions and health-care disciplines [3]. The multidisciplinary team meeting (MDTM, also called tumour board) is a key component of multidisciplinary cancer care. MDTMs are organized for certain types of cancer and bring together the clinicians who are essential for diagnosis and treatment [4]. They potentially enable interdisciplinary information exchange in order to find consensus on the best potential treatment options for one specific patient. MDTMs are viewed to have a positive impact on decision-making, team communication, and coordination within the multidisciplinary team as well as coordination with patients [4, 5]. However, MDTMs require significant investment and effective organization [6, 7]. A systematic review by Lamb et al. reveals that decisions in MDTMs are made by physicians, who primarily base their decision-making on biomedical information [7]. Other information, such as patients’ psychosocial concerns or preferences, was often unknown or neglected [8]. However, if MDTMs wished to make decisions that are consistent with patients’ preferences and goals (i.e., patient-centred decisions), information on the patient’s perspective would become an essential input into MDTM discussions [9]. A prerequisite for this is for the physicians to know the patient [10]. A recent observational study from New Zealand reported that often only one or two of the physicians at the MDTM had met the patient before [11]. Then again, studies on gastrointestinal MDTMs have shown that one of the main reasons for MDTM recommendations not being translated into action was that they were incompatible to patient preferences [12, 13]. In summary, even though MDTMs are widely utilized, evidence on the effects of MDTMs is limited [14] and little is known about the process of decision-making in MDTMs.

Another major change in modern healthcare is the shift towards a more patient-centred approach. A central dimension of patient-centred care is shared decision-making (SDM) [15]. SDM is a process in which the physician and the patient both play an active role in making decisions. Each of them shares important information from their point of view (i.e., the physician medical knowledge and the patient his or her values, preferences and goals for care) and they subsequently come to a decision that both parties can agree on [16, 17]. SDM is especially relevant in oncology, where in many cases several treatment options with different risks and benefits exist (i.e., high level of preference-sensitivity), and where treatments often have a considerable impact on the patient’s quality of life [16, 18]. Several studies have shown that patients wish to participate in the decision-making process [19]. There are policy-related activities in many countries to foster SDM [17]. Also, National Cancer Plans and practice guidelines are advocating patient-centred care and SDM [20, 21]. Besides an ethical imperative, SDM has been shown to be associated with patients being better informed, knowing more about potential risks and benefits of different options, and as a result to patients being more satisfied with the decision-making and treatment process [22]. Nevertheless, a significant gap between the interest in and well-established indication for SDM and the implementation of it in routine practice persists [23, 24].

Having established the importance of both MDTMs and SDM in cancer care, one has to ask how these two endeavours can be combined. Lamb et al. concluded in their systematic review that patient involvement in MDTMs has not often been studied [7]. Some argue that patients should be present at MDTMs [25]. However, the majority of physicians do not support this recommendation [8]. Thus, Sharma and colleagues argue for the inclusion of the patient’s perspective in the MDTM discussion through patient advocates such as specialist nurses [26]. Nurses have been found to be more open to the involvement of patient perspectives into the MDTM discussion [7]. Nonetheless, nurses and other healthcare professionals are seldom heard at MDTMs [7, 8]. Furthermore, Sharma and colleagues suggest that MDTMs recommend a range of different options [26]. These recommendations could subsequently be discussed in the patient-physician consultation. Several participants of a focus group study expressed that decisions about treatment plans cannot be finalized in an MDTM, but have to be taken back to the patient first [8]. However, most current research on decision-making in MDTMs focused on the impact of MDTMs on the recommendations made and whether these will be subsequently implemented. Previous studies were mostly carried out for specific tumours, leaving unclear whether the results can be generalized to MDTMs for different tumours. Due to the limited extant research, it is important to gain further understanding on how decisions are made in MDTMs and whether patient perspectives are represented at MDTMs. Therefore, the aim of this study was to observe MDTMs in order to gain insight into their decision-making processes and to examine how the views of patients are considered in MDTMs where patients do not attend.

## Materials and Methods

### Study design

A qualitative study was conducted using non-participant observation at multidisciplinary team meetings [27]. Qualitative research using observation methodology has been shown to be a useful approach to look into relatively new areas of research and to investigate processes in clinical care [28].

### Setting and subjects

Data collection was carried out at the University Cancer Center Hamburg (UCCH), a substructure of the University Medical Center Hamburg-Eppendorf (UKE), Germany. The UCCH is a comprehensive care and research centre including all medical departments of the UKE that are involved in diagnosis and treatment of cancer. At the UCCH 16 MDTMs for adult patients are held; most of them weekly, one twice a week, one every second week and one every fourth week. Each MDTM is associated with specific tumours, e.g. head and neck cancer or gynaecologic cancers.

### Data collection

Prior to non-participant observation, the management of the UCCH agreed to our visits at the MDTMs. The physicians responsible for each MDTM were contacted by email and informed about the non-participant observation in the MDTMs. They were also informed that the researchers would take a back seat during the observations in order to not disrupt the usual process. One of the 16 physicians responsible for the MDTMs did not reply to our email and therefore the date and place of this MDTM could not be determined. Thus, the remaining 15 MDTMs (94%) were included in the study.

In November and December 2013, two researchers (varying pairs of PH, JH, MH, and IS) attended each MDTM and recorded their observations in structured field notes, leading to two independent observations per MDTM. While 14 MDTMs were observed by two researchers each, one MDTM was observed by one researcher only (due to limited space in the meeting room). This was methodologically acceptable, as the field notes of the other 14 observed MDTMs showed strong overlap between the two observers. Thus, observations resulted in a total of 29 field-note documents from 15 MDTMs.

We recorded our observations on a form with pre-structured sections capturing the observer, time and place of the observation, a short description of the situational context, and participating individuals. The form also included a section for the observation memo. This section was left unstructured to not limit the domains of observation. During the MDTMs we took brief notes without disturbing the usual process. We then expanded our notes after the meetings had finished. In order to minimize observer bias, observers were blinded to each other’s field notes until they were completed. During data collection we met weekly to safeguard the quality of the observational process.

### Data analysis

The hand-written field notes were transcribed and imported into MAXQDA software (version 10; VERBI GmbH, Berlin, Germany). The analysis drew on principles of content analysis [29]. First, two researchers (PH and IS) read the entire set of field notes to gain an overview over the data. This first run-through revealed strong overlap between the two independent documents from the two researchers. Thus, only one of the documents was coded entirely. In the second document only additional aspects were considered. Second, during the initial coding process one researcher (PH) coded about 50% of the material using a paragraph by paragraph approach. After this phase of initial coding, comments on the material by a second researcher (IS) were compared to the established codes and the coding system was adapted. This was followed by axial coding to group the established codes into a coding system. The preliminary coding system was then discussed by two researchers (PH and IS) and adapted where necessary. In a next phase of focused coding, the remaining 50% of the material were coded by one researcher (PH) using the established coding system. Where necessary, additional codes were created and integrated into prior codings. As a last step, the coding system was once again discussed and revised (PH and IS). During the entire coding process we used memos to clarify codes and keep track of ideas and impressions during the process.

In addition to qualitative analysis, descriptive statistics were calculated.

### Ethics statement

The study was carried out in accordance with the Code of Ethics of the Declaration of Helsinki and was approved by the Ethics Committee of the Medical Association Hamburg (Germany). Written informed consent was obtained from all participants prior to participation.

## Results

### Characteristics and description of observed MDTMs

Characteristics of observed MDTMs are displayed in Table 1.

**Table 1:** Descriptive characteristics of observed MDTMs.

	Tumor stream	Duration of MDTM (in minutes)	Number of attending physicians	Number of cases in meeting	Mean duration per case (in minutes)
1	Dermatological MDTM	15.0	6	3	5.0
2	Gastrointestinal MDTM	15.0	20	5	3.0
3	Gynaecological MDTM	60.0	23	17	3.5
4	Head and neck cancer MDTM	50.0	11	12	4.2
5	Leukaemia MDTM	30.0	5	8	3.8
6	Liver and gall MDTM	52.5	14	11	4.8
7	Lymphoma and myeloma MDTM	40.0	15	8	5.0
8	Neuro-oncological MDTM	45.0	7	9	5.0
9	Non-entity specific oncological MDTM	55.0	25	10	5.5
10	Non-entity specific surgical MDTM	30.0	25	6	5.0
11	Non-entity specific multi-specialty MDTM	30.0	7	3	10.0
12	Prostate MDTM (postoperative)	15.0	22	9	1.7
13	Prostate MDTM (preoperative)	15.0	4	55	.2
14	Thorax MDTM	25.0	10	4	6.3
15	Uro-oncological MDTM	40.0	15	8	5.0
**Mean**		**34.5**	**13.9**	**11.2**	**4.5**
**(SD)**		**(15.8)**	**(7.5)**	**(12.7)**	**(2.2)**
**Range**		**15.0–60.0**	**4–25**	**3–55**	.**2–10.0**

11 out of 15 MDTMs were held in a room with approximately 50 chairs in 4 rows facing a wall onto which the electronic medical records (EMR) including scans from imaging techniques were projected. The MDTM’s recommendations were documented in the EMRs by a pre-assigned physician. In most sessions, senior physicians were seated in the front row, while junior staff sat in the back. One MDTM was held in another but similarly organized room, where the results from microscopic analysis could be shown to everyone. Three MDTMs were held in smaller rooms with fewer participants. Some of the smaller MDTMs did not document the MDTM’s recommendation in the EMR during the meeting.

Diverse team structures could be observed in the different MDTMs. Some MDTMs were characterized by a cooperative atmosphere, where physicians listened to what their colleagues had to say and interacted in a collegial way. E.g., they asked for each other’s opinion or reassured themselves by asking “Did I understand you correctly?” During these meetings, several physicians participated in the discussion and decision-making. In other MDTMs strict hierarchies could be observed. Those meetings showed a structure in which just one or very few senior physicians engaged in the discussion. In some cases tension between physicians was observed during the discussion and the atmosphere was described by the observers as “emotionally charged” (e.g., in one situation where an oncologist made a suggestion about a surgical intervention).

### Main aspects of the decision-making process in MDTMs

Results suggest that decision-making processes in MDTMs can be divided into two main aspects, a) information exchange, and b) deciding on a recommendation. These two aspects are presented in more detail below.

#### Information exchange

Physicians predominantly reported and discussed medical information. In most cases a physician briefly presented the patient’s medical history (e.g., diagnoses, comorbidities, prior treatments). During these presentations, scans from diagnostic imaging techniques were projected onto the wall. Besides medical information and the patient’s age, which was almost always mentioned, very little information was exchanged about the patient. Sometimes the patient’s general state of well-being was mentioned (e.g., “She is well. She walks around.”). In a few cases other demographic or psychosocial characteristics, such as the patient’s cultural background, his or her profession, or substance abuse by the patient were briefly stated (e.g., “He is an alcoholic and smoker.”). In very few cases the patient’s perspective or preference regarding treatment options were explicitly mentioned by presenting physicians. Familiarity with the patient and his or her case varied greatly. In some cases the attending physicians seemed to know the patient very little or not at all (e.g., none of the participants knew if a certain procedure was scheduled already).

#### Deciding on a recommendation

MDTMs decided both on further diagnostic procedures and on treatment recommendations. Physicians mainly based their decision-making on the medical information discussed. In those cases where demographic or psychosocial information was exchanged, only the patient’s age sometimes noticeably influenced the recommendations given. In those few cases, where patients’ treatment preferences were mentioned, they were mostly not taken into account in the subsequent decision-making process of the MDTM (e.g., “Then [if patient wishes not to receive chemo-therapy] you need to prevail.”). In some cases, members of the meeting even voiced their reluctance to follow a patient’s preference. Then again, there was also a minority of physicians who made an effort to include the patient’s perspective in the discussion (e.g., one physician stated the treatment preferences of the patient and his family several times).

Many of the presented cases seemed to allow several possible treatment options, rather than one clear-cut best treatment. Within the MDTM, physicians showed uncertainty regarding the diagnosis or best treatment option (e.g., a senior physician saying “My feeling is to leave it be, but it is debatable.”). At some points more than one option was discussed (e.g., a physician stating “We have two options now.”). However, almost always a single option was written down in the documented recommendation at the end of a case discussion. In three cases only, the field notes reflected that more than one option was documented following the discussion. Even though the observers had the impression that many decisions were preference-sensitive, very few physicians expressed that the patient’s preference should be included in the decision-making process.

During MDTMs some physicians mentioned past or planned physician-patient-interactions. In a couple of cases physicians mentioned that they intended to discuss the recommended treatment option(s) with the patient after the MDTM (e.g., “We should have an honest and open discussion [with the patient].”). In very few cases physicians reported having had a consultation with the patient and/or relatives about possible treatment options prior to the MDTM (e.g., “[…], as I discussed with the patient.”). In one case a physician explicitly refused to talk to the patient about treatment options, saying “Why should I talk to the patient? I don’t need to”.

Decisions in the MDTMs were made to a similar extent either by one senior or head physician alone or jointly by several senior or head physicians. Junior physicians were not observed to play a prominent role. In some cases physicians deliberated different options in the team. However, joint deliberation did not always necessarily lead to joint decision-making in the MDTM. To a small extent the MDTMs decision seemed to be mere rubber-stamping of decisions that had already been made by the presenting physician (e.g., a physician stated what he and the head physician had decided before the MDTM).

## Discussion

We found that individual patient characteristics or patient treatment preferences were rarely discussed, let alone taken into account in the subsequent decision-making processes about treatment recommendations. Physicians mainly exchanged medical information and based their decision-making largely on this information. At the same time, preference-sensitive issues were often noted by the observers, i.e. a situation in which patient preferences would be of particular importance [18]. In the few cases where patient preferences were specified, this information was rarely taken into account for the decision-making regarding the treatment recommendation. This pattern indicates a paternalistic decision-making culture in the MDTMs observed. Furthermore, the results show that in some cases joint discussion and less often joint decision-making were observed between physicians who were mostly in senior positions. Junior physicians were not found to play a significant role in the process. This influence of hierarchical structures was also found in a recent observational study on MDTMs in New Zealand [11]. Also, we did not observe other health care disciplines (e.g. nurses, psycho-oncologists) participating in MDTM discussions. This aligns with other findings [8, 30, 31]. Thus, although good teamwork has been argued to be an essential MDTM quality criterion [7], there is considerable room for more interdisciplinary communication and cooperation.

Besides hierarchical structures, the observed time pressure and setting could be possible barriers to more internal participation. Many patients had to be discussed in a short period of time. This left little room for a detailed information exchange and consideration of the patient perspective.

From the results it remains unclear how much the participating physicians knew about their patients apart from the medical information. The fact that they rarely shared other information in MDTMs does not necessarily mean that they did not know this type of information. However, they rarely explicitly took it into account when making a treatment recommendation. Previous studies revealed that the most frequent reason for not implementing MDTM recommendations was that they did not meet patient preferences [12, 13]. Thus, it is difficult to establish an individualized treatment plan and unlikely to implement it, if the professionals in an MDTM make a recommendation without explicitly considering the patient’s perspective, as found in the current study. A prerequisite for being able to include the patient’s perspective is sufficient familiarity with the patient’s views and wishes, not just the medical information. It has been argued elsewhere that other health professionals such as nurses or psycho-oncologists can be helpful as a patient advocate, who transports the patients’ wishes to MDTMs [26]. Additionally, an observational study on MDTMs found that better knowledge of the patient increased the inclusion of other health disciplines in the discussion [10]. Another possibility would be to invite the patients to participate in the MDTMs, an option advised by the accreditation guidelines for breast cancer centres in North Rhine-Westphalia, Germany [32]. However, a recent study in this area has shown that only 12% of eligible patients were asked to participate in MDTMs [33]. This suggests that guidelines alone are not sufficient to change practice.

Also, in order to achieve SDM, room in the MDTM’s discursive culture to discuss the patient’s individuality during the meetings would be needed. One component towards a culture change in the direction of an increased consideration of patient preferences in MDTMs could be trainings for health care providers. Other studies on MDTMs have also called for training in such non-technical skills [9, 34]. At the same time, current literature on SDM suggests that a change in medical culture towards more SDM is likely to take more components than just training of health care professionals: e.g. decision aids and other resources to encourage patients’ active engagement in decision making, and routine measurement of SDM to create feedback-loops and accountability [35, 36].

A main strength of this study is the investigation of a topic which has received little attention so far, despite its impact within the provision of multidisciplinary and patient-centred care. In this exploratory study, the method of non-participant observation enabled us to observe MDTMs as they happen in everyday care without the distortion self-report measures and interviews have to tackle. A main limitation of this exploratory study is the fact that it incorporates findings from only one comprehensive cancer centre in Germany. Therefore, results have limited generalizability. However, given that comprehensive cancer centres are guided by similar directives and have similar structures, we would expect to find similar results at other sites. To test this hypothesis it would be desirable to widen the scope of future studies to multiple centres and across healthcare settings. Also, finding a way to reliably identify the medical specialties taking part in the MDTM discussion, which was not possible in this study using non-participant observation, would shed light on a potentially influential variable. Furthermore, gaining insight on how MDTM recommendations were discussed with the patients after the MDTMs would considerably more the understanding of this topic. This is a crucial question, which should be investigated in future research. A large-scale international self-report study on breast cancer MDTMs revealed that physicians often see the MDTM recommendation as a “final decision” and think it has to be implemented [30]. Also, a recent interview study from the UK showed a limited junction between the MDTM and the patient [37]. Those findings suggest that there often is little room for open discussion about the recommendation with the patient after the MDTM.

In order to enhance SDM, it would be helpful, if MDTMs would not only document the recommendation of one treatment option, but rather list different options, if applicable. Lamb et al. [9] even argue that MDTMs should also document if there is considerable dissent about the best treatment option between different physicians. Further research is needed to investigate longitudinal aspects of the decision-making process in oncology, from the first contact with the patient to the MDTM until a final decision is reached.

In summary, for MDTMs and SDM to be combined, it would be essential that patient preferences are elicited before the MDTM, and that these patient preferences are included in the MDTM discussion and decision. Furthermore, MDTM recommendations (possibly more than one option) need to be taken back to the patient after the MDTM and discussed with him or her openly. This would be a step towards MDTMs taking all three pillars of evidence-based medicine (EBM) into account, i.e. the individual clinical expertise and the best external evidence in combination with the values and preferences of the informed patient [38–40].

### Conclusion

The study has shown that decision-making in MDTMs is mainly based on medical information and patient preferences receive little attention. Thus, the current structure of MDTMs in Germany serves as a barrier to the implementation of SDM. These barriers could be overcome by a range of changes. These comprise the inclusion of patient preferences in MDTM discussions, better teamwork in MDTMs, MDTM recommendations including more than one option (if applicable), and an open discussion of recommended options with the patient after the MDTM. Longitudinal research is necessary to further investigate this topic.

### Practice Implications

For MDTMs to achieve SDM and EBM, they will need to find methods of including patient and nurses perspectives in the MDTM discussion.
